# Six methods to determine expiratory time constants in mechanically ventilated patients: a prospective observational physiology study

**DOI:** 10.1186/s40635-024-00612-z

**Published:** 2024-03-07

**Authors:** Filip Depta, Caitlyn M. Chiofolo, Nicolas W. Chbat, Neil R. Euliano, Michael A. Gentile, Dušan Rybár, Viliam Donič, Marko Zdravkovic

**Affiliations:** 1 Department of Critical Care, East Slovak Institute for Cardiovascular Diseases, Ondavská 8, Košice, 040 01 Slovakia; 2 Faculty of Medicine, Pavol Jozef Šafarik University, Košice, Slovakia; 3 Quadrus Medical Technologies, Inc, White Plains, NY USA; 4 Convergent Engineering, Gainesville, FL USA; 5 Department of Anesthesia, Duke University Medical Center, Durham, NC USA; 6 Department of Physiology, Pavol Jozef Šafarik University, Košice, Slovakia; 7 Faculty of Medicine, University of Ljubljana, Ljubljana, Slovenia; 8 Department of Anaesthesiology, Intensive Care and Pain Management, University Medical Centre Maribor, Maribor, Slovenia

**Keywords:** Expiratory time constant, Mechanical ventilation, Exhalation, Volume-controlled ventilation, Pressure-controlled ventilation

## Abstract

**Background:**

Expiratory time constant (*τ*) objectively assesses the speed of exhalation and can guide adjustments of the respiratory rate and the I:E ratio with the goal of achieving complete exhalation. Multiple methods of obtaining *τ* are available, but they have not been compared. The purpose of this study was to compare six different methods to obtain *τ* and to test if the exponentially decaying flow corresponds to the measured time constants.

**Methods:**

In this prospective study, pressure, flow, and volume waveforms of 30 postoperative patients undergoing volume (VCV) and pressure-controlled ventilation (PCV) were obtained using a data acquisition device and analyzed. *τ* was measured as the first 63% of the exhaled tidal volume (V_T_) and compared to the calculated *τ* as the product of expiratory resistance (R_E_) and respiratory system compliance (C_RS_), or *τ* derived from passive flow/volume waveforms using previously published equations as proposed by Aerts, Brunner, Guttmann, and Lourens. We tested if the duration of exponentially decaying flow during exhalation corresponded to the duration of the predicted second and third *τ*, based on multiples of the first measured *τ*.

**Results:**

Mean (95% CI) *measured τ* was 0.59 (0.57–0.62) s and 0.60 (0.58–0.63) s for PCV and VCV (p = 0.45), respectively. *Aerts* method showed the shortest values of all methods for both modes: 0.57 (0.54–0.59) s for PCV and 0.58 (0.55–0.61) s for VCV. *Calculated* (C_RS_ * R_E_) and *Brunner’s τ* were identical with mean* τ* of 0.64 (0.61–0.67) s for PCV and 0.66 (0.63–069) s for VCV. Mean *Guttmann’s τ* was 0.64 (0.61–0.68) in PCV and 0.65 (0.62–0.69) in VCV. Comparison of each *τ* method between PCV and VCV was not significant. Predicted time to exhale 95% of the V_T_ (i.e., 3*τ) was 1.77 (1.70–1.84) s for PCV and 1.80 (1.73–1.88) s for VCV, which was significantly longer than measured values: 1.27 (1.22–1.32) for PCV and 1.30 (1.25–1.35) s for VCV (*p* < 0.0001). The first, the second and the third measured *τ* were progressively shorter: 0.6, 0.4 and 0.3 s, in both ventilation modes (*p* < 0.0001).

**Conclusion:**

All six methods to determine *τ* show similar values and are feasible in postoperative mechanically ventilated patients in both PCV and VCV modes.

**Supplementary Information:**

The online version contains supplementary material available at 10.1186/s40635-024-00612-z.

## Introduction

Expiratory time constant (*τ*) is an important, yet not widely used parameter providing information about the mechanical properties of the respiratory system. Although not routinely used in clinical practice, *τ* can be used to optimize mechanical ventilation by predicting time for complete exhalation, optimizing the respiratory rate, calculating common inspiratory variables without end-expiratory pause or assessing the response to bronchodilators [1–3]. Further, it has been integrated into complex algorithms of supported modes of ventilation to achieve optimal targeting schemes, such as adaptive support ventilation and adaptive mechanical ventilation [4, 5].

Commonly studied parameters of lung mechanics, such as respiratory system compliance (C_RS_), resistance (R_RS_), driving pressure (dP) or plateau pressure (P_PLAT_), are routinely obtained during inspiration. However, such parameters are influenced by ventilator settings (i.e., inspiratory flow, pressure, tidal volume [V_T_], and inspiratory time). Exhalation, on the other hand, is usually passive and therefore expiratory variables, such as *τ*, provide a more independent measure of lung mechanics compared to inspiratory parameters.

*τ* has been traditionally defined as the product of C_RS_ and R_RS_ in a single compartment lung model during passive deflation [6, 7]. However, it is better defined as the amount of time that an exponentially decaying quantity takes to decay by a factor of 1/e, where the first *τ* represents 63% of exhaled V_T_, 2*τ* 86%, 3*τ* 95%, 4*τ* 98% and *5τ* represent 99% of the exhaled V_T_ etc. Accordingly, all time constants should be of equal duration in an exponentially decaying flow [8].

Several authors have studied *τ* in heterogenous patient populations using different methodologies [7–13]. For example, calculated *τ* may adopt either dynamic or static C_RS_ and inspiratory or expiratory airway resistance. On the other hand, some authors do not use quasi-static variables and rely on direct measurements of *τ* from the expiratory flow curve [10, 13]. Therefore, different formulations are often used interchangeably in clinical practice and may be a source of imprecision when interpreting results.

Comparisons of these different methods of *τ* determination had not been performed. Therefore, the aim of this prospective observational study was to compare measured *τ* from the expiratory flow waveform (i.e., representing the gold standard), with five alternative methods of *τ* calculation under variable flow and constant flow ventilation (i.e., pressure-controlled ventilation [PCV] and in volume-controlled ventilation [VCV]) in routine clinical care of patients following cardiac surgery. This study tested whether lung emptying can be accurately described (and also predicted) by a single ***τ*** value (i.e., explored whether the time constant can be used to characterize the exponentially decaying flow during passive exhalation). Comparison of the six methods of* τ* determination would enable identifying the most reliable approach for assessing *τ* at the bedside and making more informed decisions on setting mechanical ventilation parameters based on *τ*.

## Materials and methods

### Study design and participants

This prospective observational study was performed in a tertiary referral university hospital (East Slovak Institute for Cardiovascular Diseases, Kosice, Slovakia) from December 2022 to February 2023 and conforms to the relevant STROBE reporting guidelines. The institutional ethics committee approved the study (IEC N.A3112022) and informed consent was obtained from all patients prior to enrollment. The study was retrospectively registered at ClinicalTrials.gov (NCT05827640). Institutional ethics committee also confirms that all methods and experimental protocols were carried out in accordance with relevant guidelines and regulations.

Thirty adult patients undergoing elective cardiac surgery with extracorporeal circulation were included. Patients were excluded from the study if they had any known lung disease or previous thoracic surgery. After surgery, all patients were transferred to the intensive care unit (ICU) and supported with mechanical ventilation (Servo-U, Maquet, Getinge AB, Solna, Sweden).

### Mechanical ventilation parameters and data acquisition

First, the ventilation mode was set to mandatory VCV for 15 min. Next, mandatory PCV was set for another 15 min. All patients were in the supine position, sedated and paralyzed using continuous infusion of propofol and atracurium with no spontaneous breathing efforts. During VCV, an end-inspiratory pause (T_PAUSE_ 10%) had been added to obtain inspiratory P_PLAT_ under static conditions. ARDSNet tables were used to determine predicted body weight (PBW) for all patients [14].

Respiratory variables in VCV for all patients were set as follows: positive end-expiratory pressure (PEEP) of 6–9 cmH_2_O depending on the local protocol, protective V_T_ of 7 mL/kg PBW, T_PAUSE_ as 10%, I:E ratio of 1:2, rise time 5% and the respiratory rate of 14 breaths/min. After 15 min, VCV was changed to PCV with the same ventilator settings and inspiratory pressure was set in a way to best match the V_T_ during VCV.

**Fig. 1:**
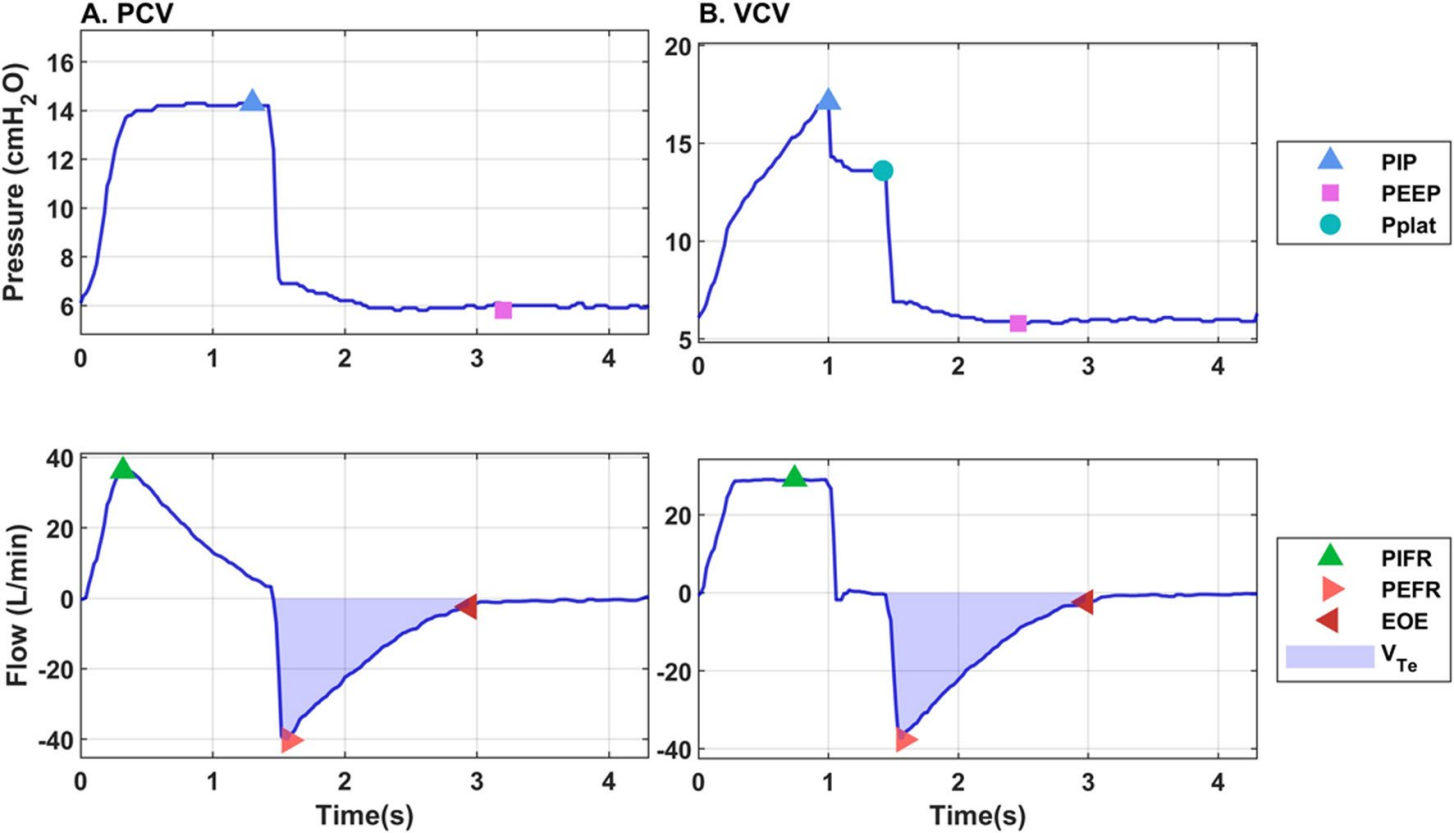
Pressure and flow waveforms during **A** pressure-controlled ventilation (PCV) and **B** volume-controlled ventilation (VCV)**.** Measurements extracted from the waveforms and used in calculations of the expiratory time constant (*τ*) are highlighted. *EOE* end of exhalation (0.04 L/s), *PEEP* positive end-expiratory pressure, *PEFR* peak expiratory flow rate, *PIP* peak inspiratory pressure, *P*_*PLAT*_ plateau pressure, *V*_*Te*_ exhaled tidal volume

Pressure, flow, and volume data were recorded at 20 ms sampling rate after admission to the ICU using a data acquisition device (DR WAVE®, Quadrus Medical Technologies, NY, USA) connected to the Servo-U ventilator using Servo-U flow sensor. All results in the study were derived from waveforms recorded by the device and extracted measurements and calculations included: peak inspiratory pressure (PIP), P_PLAT_, PEEP, exhaled V_T_, inspiratory airway resistance (R_I_), expiratory airway resistance (R_E_), C_RS_, peak inspiratory flow, peak expiratory flow rate (PEFR), and expiratory flow at 50% and 75% of the exhaled V_T_ as appropriate. Data were visualized and analyzed using Matlab® R2021b. An example of the pressure and flow waveforms for a single breath in PCV and VCV is depicted in Fig. 1.

### Methods of τ calculation and measurement

Six different methods were used to evaluate the expiratory *τ*. We have defined the start of exhalation (SOE) using the breath phase flag from the Servo-U ventilator, which corresponded to the first zero-flow crossing, and the end of exhalation (EOE) as the first time when the expiratory flow decreased to 0.04 L/s from PEFR, which also reduced the signal noise and artifacts caused by cardiac oscillations [13].

Exhaled V_T_ was used in all formulae. Expiratory flows were treated as positive values throughout. *τ* was measured, calculated or derived from expiratory flow curves as follows:

1. Measured *τ* was derived from the expiratory flow waveform [1, 9]. Flow was integrated from SOE to EOE using the trapezoidal rule to obtain volume expired in time. A mathematical algorithm was created to measure time from SOE until 63% of the exhaled V_T_ was reached (*τ*_*1*_). Similarly, to obtain the second *τ* (*τ*_*2*_) and the third *τ* (*τ*_*3*_), the time durations from 63 to 86% of the exhaled V_T_ and from 86 to 95% of the exhaled V_T_ were measured, respectively.

2. Calculated *τ* as the product of compliance and resistance [7]. Compliance and R_E_ were calculated using the respective formulas for PCV and VCV. R_E_ was calculated as proposed by Jonson [8]:

$$\tau ={R}_{E}\cdot {C}_{RS}$$

For PCV: $${R}_{E}=\frac{PIP- PEEP}{PEFR}$$,$${C}_{DYN}=\frac{{V}_{T}}{PIP-PEEP}$$

For VCV: ${R}_{E}=\frac{{P}_{PLAT} -PEEP}{PEFR}$,${C}_{STAT}=\frac{{V}_{T}}{{P}_{PLAT}-PEEP}$ where C_STAT_ is static compliance and C_DYN_ is dynamic compliance, and PEFR is peak expiratory flow rate.

3. $\tau$ calculated with the Aerts formula [10].

$\tau =\frac{{0.5 \cdot V}_{T}}{{\dot{V}}_{50}- {\dot{V}}_{end-exp}}$, where ${\dot{V}}_{50}$ is expiratory flow at 50% of expired V_T_ and ${\dot{V}}_{end-exp}$ expiratory flow at end-expiration.

4. $\tau$ calculated with the Brunner formula [11].

$$\tau =\frac{{V}_{T}}{PEFR}$$

**Fig. 2:**
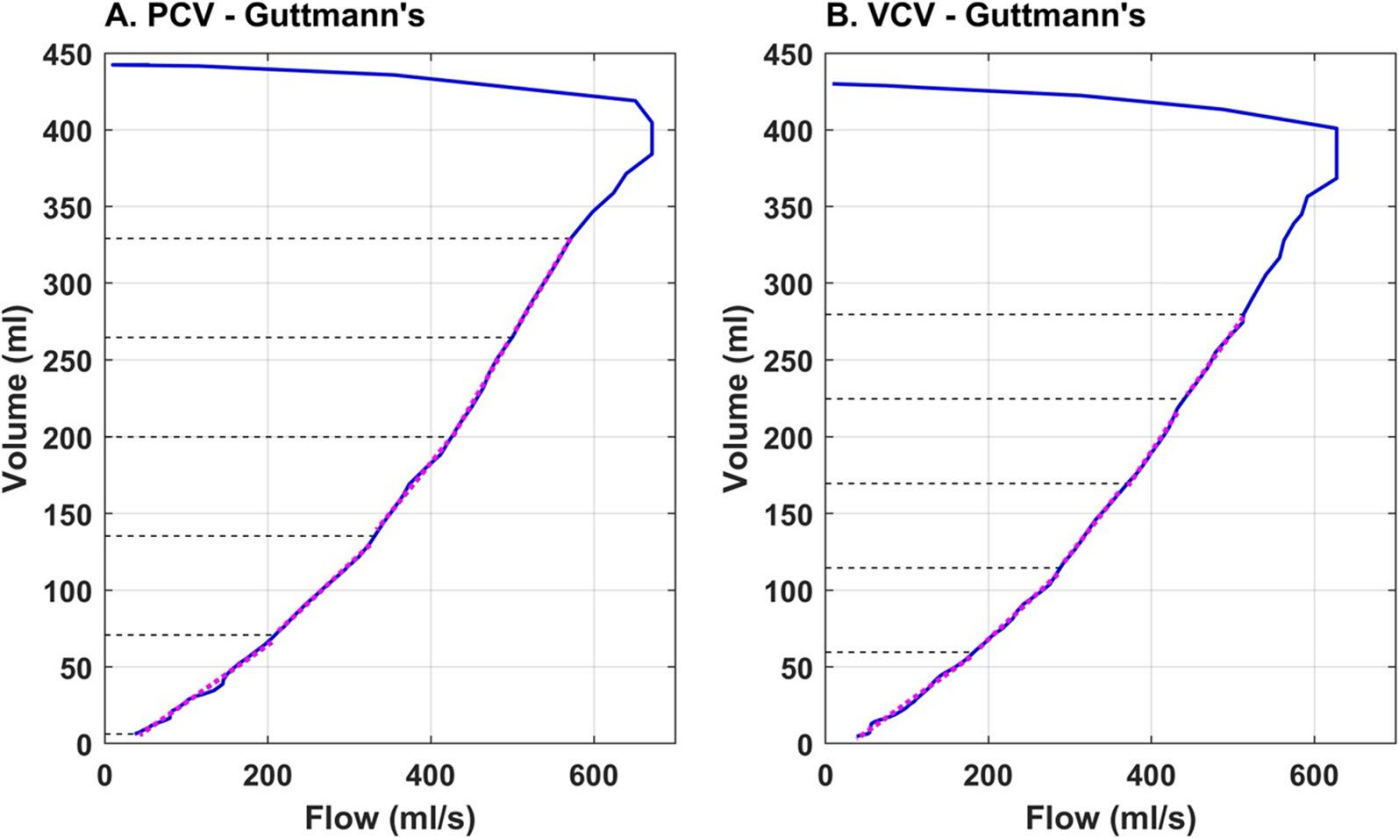
Guttmann’s method for determining the expiratory time constant (*τ*) in **A** pressure-controlled ventilation (PCV) and **B** volume-controlled ventilation (VCV)

5. $\tau$ calculated with the Guttmann formula [12]. The expiratory volume versus flow curve was divided into five equal volume slices from the maximum slope in the expiratory flow curve following the PEFR, to the end of exhalation (Fig. 2). *τ* was calculated for each slice of the volume versus flow curve using the least squares fitting method. Final *τ* was then obtained as the average of the *τ* from all five slices.

6. $\tau$ calculated with the Lourens formula [13].

$\tau =\frac{{0.75 \cdot V}_{T}}{{\dot{V}}_{75}- {\dot{V}}_{end-exp}}$, where ${\dot{V}}_{75}$ is expiratory flow at 75% of the exhaled V_T_ (i.e., 25% expired volume, or 75% of the volume remaining to be exhaled), ${\dot{V}}_{end-exp}$ is expiratory flow at end expiration.

**Fig. 3:**
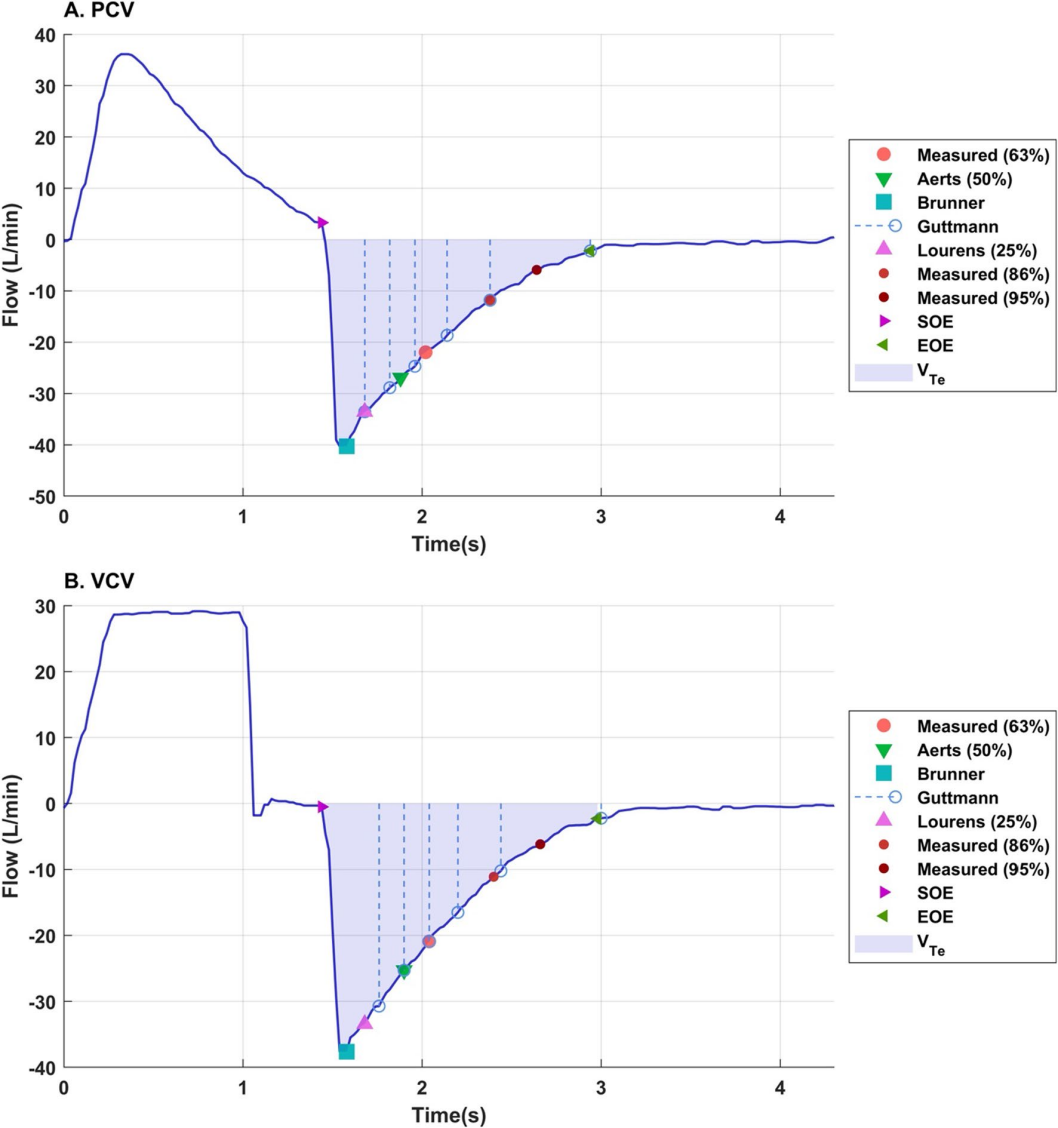
Flow waveform during **A** pressure-controlled ventilation (PCV) and **B** volume-controlled ventilation (VCV) highlighting points used in different methods to obtain the expiratory time constant (*τ*). *EOE* end of exhalation, *Meas.* measured, *V*_*Te*_ exhaled tidal volume

The details of *τ* determination as per each method are also shown graphically in Figs. 1, 2, 3.

The *τ* by each method was computed for each patient-breath and averaged over all PCV or VCV breaths. The mean *τ* was then taken across all patients. Measured *τ* was taken as the *reference* for comparison with each of the other five methods.

### Outcomes and definitions

The primary outcome of this study was to compare the differences between measured *τ*_*1*_ and five different methods of calculating *τ* in passive, mechanically ventilated patients under variable flow (PCV) and constant flow (VCV) conditions.

The secondary outcomes were: (1) comparison of the *τ* between PCV and VCV for each method of *τ* determination; (2) comparison of the predicted time to exhale 95% of the expired V_T_ using the first measured *τ* multiplied by 3 (i.e., 95% of the exhaled V_T_ = 3*τ_*1*_) with the measured time to exhale 95% of the V_T_; (3) comparing the duration of the first, the second and the third measured *τ* (i.e., *τ*_*1*_, *τ*_*2*_, and *τ*_*3*_, respectively), as per exponentially decaying *τ* equation.

### Statistical analysis

Categorical data are expressed as n (%), continuous data are expressed as mean or median with 95% confidence intervals (95% CI). The distributions were tested for normality using Kolmogorov–Smirnov test and for skewness and kurtosis. Comparison of the six *τ* means over all breaths and patients was performed using one-way ANOVA. Here, each method of *τ* determination was considered independent, as the statistical tests are more robust in this case (i.e., larger differences between means are needed when compared with repeated measures t-tests for the same level of statistical significance). If significant, then Dunnett’s post hoc test was used to compare each of the five methods of *τ* determination with the measured *τ* as the reference. Student’s t test was used to compare mean *τ* between PCV and VCV within each method of *τ* determination. Student’s t test was also used to compare the first measured *τ* multiplied by 3 with the measured time required to expire 95% of the exhaled V_T_. In comparing the first, the second, and the third mean measured *τ*, the Welch’s one-way ANOVA was followed by the Games–Howell post hoc test. P values lower than 0.05 were considered statistically significant. Statistical tests were run using Python 3.11.0 SciPy version 1.10.0 and R (The R-foundation for Statistical Computing, Vienna, Austria), and visualizations were performed using graphical user interface for R (RStudio version 4.3.2).

## Results

Thirty patients were included in this prospective observational study. Their median (IQR) age was 65 (62–68) years, BMI was 29 (28–30) kg/m^2^ and 23 (67%) was male. Other patient characteristics are summarized in Additional file 1: Table S1. The respiratory variables are presented in Table 1. The mean number of recorded breaths per patient was 215 (95% CI 209–221) for PCV mode and 211 (95% CI 203–219) for VCV mode and they were analyzed via six different methods to obtain the *τ*.

**Table 1:** Respiratory variables before the beginning of the 15-min ventilation recordings, means with 95%CI are presented

	PCV (*n* = 30)	VCV (*n* = 30)
V_T_ (mL/kg/PBW)	7.1 [7.0–7.1]	7.1 [7.0–7.1]
RR (breaths/min)	14	14
MV (l/min)	6.7 [6.3–7.1]	6.6 [6.3–7.0]
PEEP (cmH_2_O)	7.0 [6.7–7.4]	7.0 [6.6–7.4]
PIP (cmH_2_O)	16 [15–17]	19 [18–20]
P_PLAT_ (cmH_2_O)	N/A	16 [15–17]
dP (cmH_2_O)	N/A	8.7 [8.2–9.2]
C_RS_ (mL/cmH_2_O)	48 [44—51]	52 [48—56]
R_**I**_ (cmH_2_O/L/s)	14.2 [11.8–18.4]	6.8 [5.6–8.8]
R_**E**_ (cmH_2_O/L/s)	13.7 [13.1–14.3]	12.9 [12.3–13.5]

*C*_*RS*_ respiratory system compliance, *dP* driving pressure, *MV* minute ventilation, *n* number, *N/A* not applicable, *PBW* predicted body weight, *PCV* pressure-controlled ventilation, *PEEP* positive end expiratory pressure, *PIP* peak inspiratory pressure, *P*_*PLAT*_ inspiratory plateau pressure, *RR* respiratory rate, *R*_***I***_ inspiratory airway resistance, *R*_***E***_ expiratory airway resistance, *VCV* volume-controlled ventilation, *V*_*T*_ tidal volume. C_RS_, R_**I**_ and R_**E**_ values were computed from recorded waveforms using data acquisition device (DR WAVE®, Quadrus Medical Technologies, NY, USA). Values are displayed as means/medians with 95% confidence intervals

**Fig. 4:**
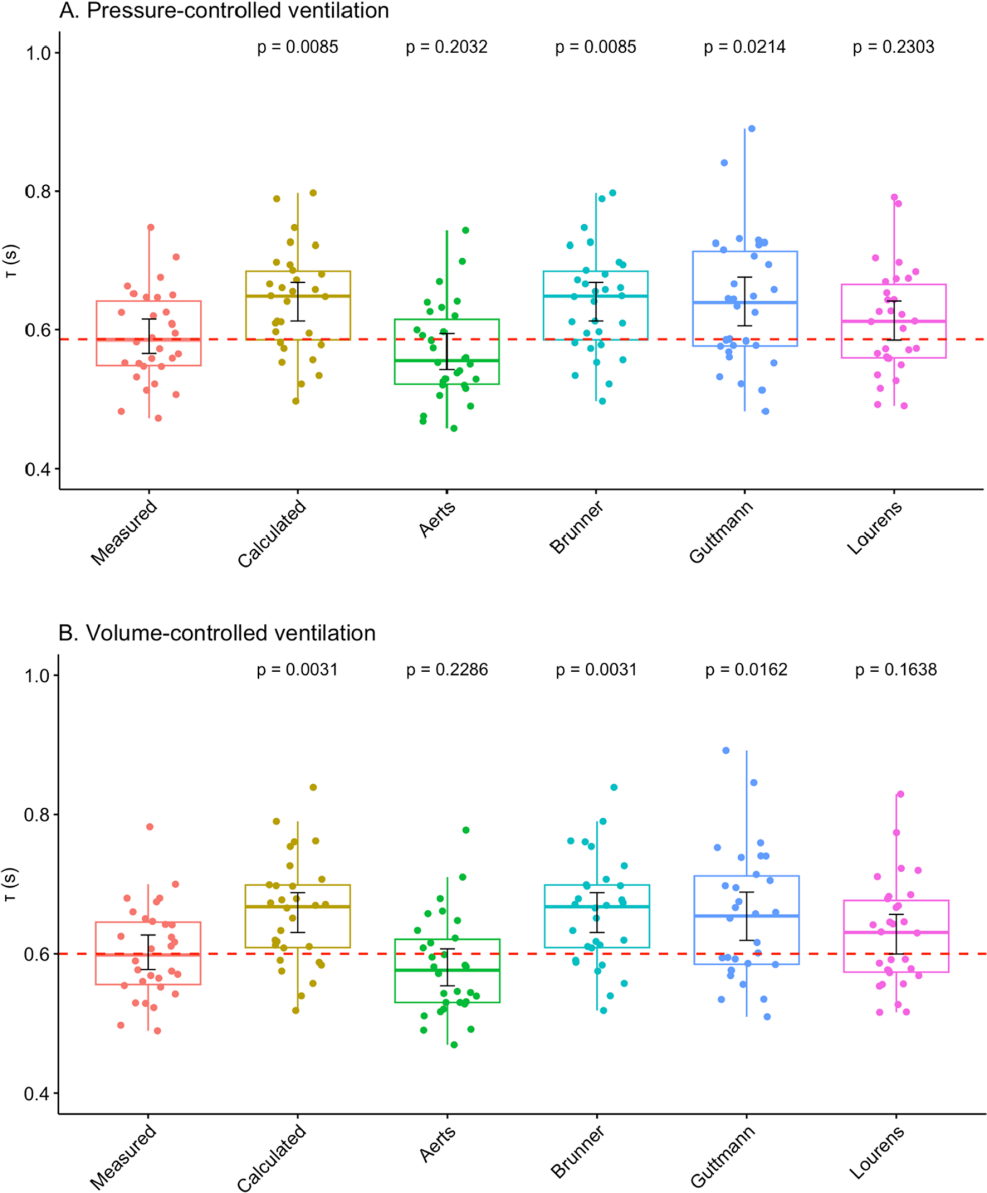
Boxplots showing comparison of measured first expiratory time constant (*τ*_*1*_) with each of the other five methods of *τ* calculation in: **A** pressure-controlled ventilation (PCV) and **B** volume-controlled ventilation (VCV). Values are displayed as means, IQRs and 95% confidence intervals (black cursors within the boxplots). Mean of the measured *τ*_*1*_ is represented as the dashed red line. The five methods of *τ* determination were compared with the measured *τ*_*1*_ as the reference using the Dunnett’s post hoc test, displaying adjusted p values

Mean values of *τ* for each method are shown in Table 2 where the *τ* as determined by each method during PCV *versus* VCV was compared, with no significant differences between the modes. The comparison of each *τ* method to the first measured value of *τ (τ*_*1*_) as the "gold standard" was then performed and is presented in Fig. 4 for each ventilation mode. *Brunner*, *Calculated as C*_RS_*R_*E*_, and *Guttmann τ* were all significantly different from the measured *τ*_*1*_ in both ventilation modes (overall ANOVA: *p* < 0.001, for PCV and *p* < 0.0001 for VCV). Mean differences between measured *τ*_*1*_ and other 5 methods to determine *τ* in PCV and VCV for each individual patient are available in the Additional file 2: Table S2 and Additional file 3: Table S3.

**Table 2:** Six methods to determine the expiratory time constant (*τ*) in pressure-controlled ventilation (PCV) and volume-controlled ventilation (VCV)

	PCV	VCV	*p*
Measured *τ*_*1*_ (s)	0.59 (0.57–0.62)	0.60 (0.58–0.63)	0.45
Calculated *τ* (s)	0.64 (0.61–0.67)	0.66 (0.63–069)	0.17
Aerts *τ* (s)	0.57 (0.54–0.59)	0.58 (0.55–0.61)	0.37
Brunner *τ* (s)	0.64 (0.61–0.67)	0.66 (0.63–0.69)	0.17
Guttmann *τ* (s)	0.64 (0.61–0.68)	0.65 (0.62–0.69)	0.28
Lourens *τ* (s)	0.61 (0.58–0.64)	0.63 (0.60–0.66)	0.23

Values are shown in seconds as means with their corresponding 95% confidence intervals. Student’s t test was used to compare mean *τ* between PCV and VCV within each method of *τ* determination

**Fig. 5:**
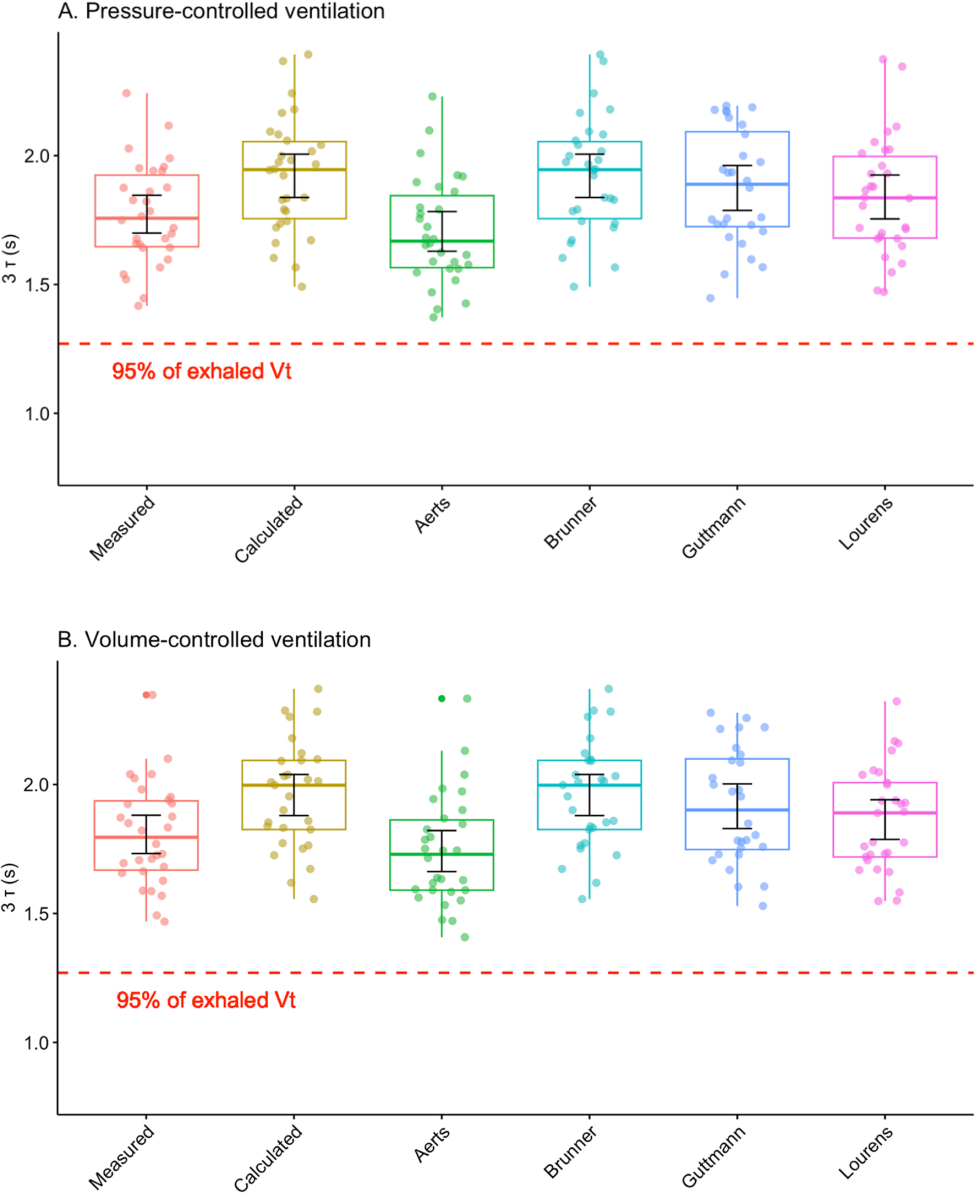
Measured time to reach the 95% of the exhaled V_T_ (red dotted line) compared with predicted times to reach 95% of the exhaled V_T_ according to each of the six methods of the expiratory time constant (*τ*) determination (3* τ). The *τ* as determined by each method was multiplied by 3 in: **A** pressure-controlled ventilation (PCV) and **B** volume-controlled ventilation (VCV). Values are displayed as means with 95% confidence intervals

To test the assumption of the time constants’ equalities, the time required to exhale 95% of the expired V_T_ was compared with the first *τ* multiplied by three (i.e., 95% of the expired *V*_T_ = 3*τ_*1*_). Predicted time to exhale 95% of the expired V_T_ was significantly different from the measured value (*p* < 0.0001). All methods overestimated the time needed to exhale 95% of the expired V_T_ (Fig. 5).

The absolute difference between predicted and measured time to exhale 95% V_Te_ was 0.5 s for both ventilation modes (95% CI 0.48–0.52 s, *p* < 0.0001 for PCV and 0.48– 0.53 s, *p* < 0.0001 for VCV) (Table 3). The time difference between predicted and measured time to exhale 95% of V_Te_ (0.5 s for both modes) represents 18% of total expiratory phase of respiratory cycle. We found that 2.2*τ_*1*_, rather than 3*τ_*1*_ are needed to exhale 95% of the V_Te_.

**Table 3:** Measured and predicted time for complete exhalation

	PCV	VCV
Complete exhalation time (s)*	1.57 (1.50–1.62)	1.60 (1.54–1.66)
Measured time to exhale 95% of V_Te_ (s)**	1.27 (1.22–1.32)	1.30 (1.25–1.35)
Predicted time to exhale 95% of V_Te_***	1.77 (1.70–1.84)	1.80 (1.73–1.88)

Values are given in seconds as means with their corresponding 95% confidence intervals

^*^Measured from start of exhalation (SOE) until the expiratory flow dropped to 0.04L/sec

^**^Measured time from SOE until exhaled tidal volume (V_Te_) reached 95%

^***^Predicted time to exhale 95% of V_T_ by multiplying the first measured (*τ*_*1*_) by three (i.e., 3*τ_*1*_)

### Time constant inequalities

The computer algorithm measured the first *τ* (*τ*_*1*_), the second *τ* (*τ*_*2*_), and the third *τ* (*τ*_*3*_) corresponding to the time needed from 0 to 63%, from 63 to 86% and from 86 to 95% of the exhaled V_T_, respectively. These three time constants were significantly different from each other (ANOVA; p < 0.0001, for both modes of ventilation) (Table 4).

**Table 4:** The first, the second, and the third *measured* expiratory time constants (*τ*_*1*_, *τ*_*2*_, and *τ*_*3*_, respectively) in pressure-controlled ventilation (PCV) and volume-controlled ventilation (VCV)

	Measured* τ*_*1*_ (s)	Measured *τ*_*2*_ (s)	Measured *τ*_*3*_ (s)	p* τ_*1*_ vs. *τ*_*2*_	p** τ_*2*_ vs. *τ*_*3*_	p*** τ_*1*_ vs. *τ*_*3*_
PCV	0.59 (0.57–0.62)	0.39 (0.37–0.40)	0.29 (0.28–0.31)	< 0.0001	< 0.0001	< 0.0001
VCV	0.60 (0.58–0.63)	0.39 (0.38–0.41)	0.30 (0.29–0.31)	< 0.0001	< 0.0001	< 0.0001

Data are shown as means with 95% confidence intervals

^*^comparison between *τ*_*1*_ and *τ*_*2*_

^**^comparison between the *τ*_*2*_ and *τ*_*3*_

^***^comparison between *τ*_*1*_ and the *τ*_*3*_

Individual per-patient differences in *τ*_*1*_,* τ*_*2*_, and *τ*_*3*_ for both modes are shown in Additional file 4: Table S4.

## Discussion

The main result of this study is that measured ***τ***, as well as the other five methods, reliably determines the expiratory time constant in postoperative patients under constant or variable flow ventilation. Regardless of ventilation mode, ***τ*** calculated as the product of R_E_ * C_RS_, Brunner, and Guttman were all significantly longer than measured ***τ***, while Aerts was shorter. Furthermore, the first, the second, and the third measured *τ* differ significantly in duration. Predicted values extrapolated from the first *τ* (3*τ) overestimated measured time to exhale 95% of the V_Te_ in all six methods in both ventilation modes. Moreover, we found that instead of 3*τ_***1***_, only 2.2*τ are needed to exhale 95% of V_Te_.

The expiratory time constant describes ideal lung emptying in a single compartment model with exponentially decreasing flow, where all time constants (equal in duration) are responsible for different volumes of exhaled air. Using the model definition, most authors have proposed to calculate the time constant instead of measuring it [6–8]. However, this model oversimplifies lung dynamics, which vary greatly in clinical practice due to complexity of the respiratory system under mechanical ventilation, especially in the presence of diverse underlying lung pathology. With changes in airway resistance during tidal ventilation and the presence of fixed additional resistances (such as the endotracheal tube—ETT), lung emptying is heavily modified compared to the simple one-compartment model. As a result, the time constants (first, second, third, etc.) can hardly be of equal duration. In our study on postoperative patients with generally favorable lung mechanics, we confirmed that the first three time constants do not equal but differ significantly indicating the single compartment model may not best represent passive exhalation in this patient group. Differences between consequent time constants can be even more pronounced in acute respiratory failure with time constant inequalities [15].

Brunner’s, Aerts’, and Lourens’ methods all use ratio of exhaled volume to either PEFR or a two-point difference of the expiratory flow waveform during later stages of exhalation, assuming a linear flow–volume relationship. However, exhalation was later found to be a rather dynamic process in mechanically ventilated patients with changing resistance [16]. Aerts’, as well as Lourens’ formulae, does not consider the early (most dynamic and resistance-dependent) part of exhalation, but only considers the more linear portion, where the single compartment model assumptions could hold and the flow-dependent resistance of the ETT was less important. Despite these physiological differences in the methods, our results show that all methods yield very similar ***τ*** in postoperative patients without prior lung disease. Further studies are needed to confirm our findings in ventilated patients with obstructive lung disease.

Lourens et al. also compared calculated ***τ*** multiplied by 3 with the measured time to reach complete exhalation as dictated by the 0.04 L/s cutoff of exhaled flow and found that mean calculated ***τ*** correlated well with real measured time to reach complete exhalation in chronic obstructive pulmonary disease (COPD) patients. We did not confirm these findings in postoperative patients without expiratory flow limitation, which is the hallmark of patients with severe COPD. This is also supported by significantly shorter ***τ*** in our cohort (***τ*** = 0.59 s) versus COPD patients (***τ*** = 2.8 s) [13]. Patients with expiratory flow limitation tend to have more linear decay of expiratory flow. It is therefore probable that Lourens’ method to determine ***τ*** may be more appropriate in patients with COPD.

Guttmann et al. proposed to calculate ***τ*** for each of five equal volume slices with the least square fitting method and found that consecutive volume portions were exhaled at nearly identical time constants in ARDS patients. Guttmann also found that resistance of the ETT represents the major resistance responsible for longer ***τ*** compared to the time constants of the pure respiratory system alone [12]. While Guttmann looks at five discrete windows of exhalation, measured ***τ*** differs in that it measures time until a certain volume is exhaled, which implicitly includes the dynamics of the flow. Measured ***τ*** were significantly shorter toward the end of exhalation (progressive shortening of subsequent ***τ***), perhaps in part due to negligible resistance of the ETT in the later parts of the flow curve [18, 19].

While ***τ*** can also be calculated as a product of C_RS_ and R_RS_, the resulting ***τ*** depends greatly on the type of compliance (C_DYN_ or C_STAT_) and resistance (R_**I**_ or R_**E**_) being used in calculations. It was proposed that at least six methods exist to determine R_**E**_, producing significantly different results [22]. Therefore, resultant ***τ*** will also be different. In this study, we achieved the most consistent results with R_**E**_ calculation using Jonson formula [22] for both modes of ventilation, where difference in pressure (P_PLAT_—PEEP in VCV or PIP-PEEP in PCV) was calculated and then divided by PEFR. Interestingly, ***τ*** calculated as the product of C_RS_ (using expiratory volume) and R_E_ using Jonson’s formula reduces to Brunner (V_Te_/PEFR). Hence, these two methods then yield the same results. Mean values for measured and calculated ***τ*** in our study differed by around 50 ms in PCV and 60 ms in VCV. Calculating ***τ*** reliably usually necessitates obtaining quasi-static variables (i.e., end inspiratory and end expiratory hold), while measuring ***τ*** directly does not. Therefore, measuring ***τ*** eliminates the need for such maneuvers, while still providing consistent results that are clinically acceptable.

Only one (or the first) ***τ*** is measured or calculated in clinical practice and routinely displayed on modern mechanical ventilators. This means that prediction of near-complete exhalation and therefore the respiratory rate is dependent on a single variable. Based on our data, the *τ* definition is not upheld in postoperative mechanically ventilated patients because the first ***τ*** multiplied by 3 does not equal complete exhalation but overestimates it significantly. Despite our findings contradicting the predictions based on exponential decay in flow, *τ* seems to remain the only variable objectively assessing the speed of exhalation in clinical practice. Significant differences between measured, calculated, or derived *τ* found in this study may be of clinical importance to properly set the respiratory rate [1]. This is likely clinically negligible in healthy lungs or at low respiratory rates, but may become significant with high respiratory rates needed to maintain adequate gas exchange. In the era of protective low tidal volume, high respiratory rates are often needed. Therefore, a correction factor is probably required if the first measured ***τ*** is used to predict the respiratory rate because we found the first ***τ*** to be the longest, while the second and the third *τ* are progressively shorter.

This physiologic study has several limitations. The main limitation is the patient selection. We have studied the ***τ*** in passive patients following cardiac surgery without known previous lung disease. Therefore, the extrapolation of our findings to other groups of patients with lung disease might not be appropriate. It is imperative that further studies on ***τ*** focus on patients presenting with restrictive and obstructive exhalation patterns. To best reflect routine clinical care at the bedside, P_PLAT_ was determined using only brief end-expiratory pause (i.e., T_PAUSE_ = 10%). By study design, we decided to average ***τ*** in all 200 breaths and therefore proper end inspiratory pause (i.e., 4 s) that is conventionally used to reliably determine P_PLAT_ could not be applied for each breath. It can therefore be assumed that calculated time constants using C_RS_ may have been slightly different if longer end-inspiratory pause was used. Similarly, equations to determine C_DYN_ and R_**E**_ in PCV provide only a rough estimation of pulmonary mechanics. Nonetheless, calculated ***τ*** in both ventilation modes are in good agreement with the other five methods.

To conclude, although differences between measured and calculated/derived ***τ*** were found, all six methods seem to reliably determine ***τ*** in postoperative patients under constant and variable mechanical ventilation. Our results challenge the time constant concept of equal ***τ*** of an exponentially decaying flow in a single compartment model. Alternatively, a search for a new variable to objectively assess the speed of exhalation with the general aim of accurately predicting time for complete exhalation is warranted. This would in turn contribute to more personalized setting of the respiratory rate consequently minimizing dynamic hyperinflation and possibly protect the lungs from injurious mechanical ventilation.

### Supplementary Information

**Additional file 1: Table S1**. Baseline patients characteristics (n = 30). ICU, intensive care unit. Values are displayed as means/medians with 95% confidence intervals or number (n) with proportion (%).

**Additional file 2: Table S2.** Per patient differences between measured τ (τ1) and 5 other methods in Pressure-controlled ventilation. Results are shown in seconds (s).

**Additional file 3: Table S3.** Per patient differences between measured τ (τ1) and 5 other methods in Volume-controlled ventilation. Results are shown in seconds (s).

**Additional file 4: Table S4**. Measured first (***τ***_***1***_), second (***τ***_***2***_) and third (***τ***_***3***_) τ in PCV and VCV.

## Data Availability

Any data-related questions should be directed to the corresponding author and are available on reasonable request.
