# Supplementary material for: Six methods to determine expiratory time constants in mechanically ventilated patients: a prospective observational physiology study
Source: Intensive Care Med Exp. 2024 Mar 7;12:25. doi: 10.1186/s40635-024-00612-z (PMC10920606; doi:10.1186/s40635-024-00612-z)
Supplement: Supplementary file 1 — Additional file 1: Table S1. Baseline patients characteristics (n = 30). ICU, intensive care unit. Values are displayed as means/medians with 95% confidence intervals or number (n) with proportion (%). [file 40635_2024_612_MOESM1_ESM.docx]

**Additional Table 1**: Baseline patients characteristics (n = 30). ICU, intensive care unit. Values are displayed as means/medians with 95% confidence intervals or number (n) with proportion (%).

| Age (years) | 65 [62-68] |
| --- | --- |
| Gender – male – n (%) | 23 (67) |
| Body mass index (kg.m^-2^) | 29 [28-30] |
| **Medical History** |  |
| Hypertension – n (%) | 25 (83) |
| Diabetes – n (%) | 15 (50) |
| Hypercholesterolemia – n (%) | 22 (73) |
| Chronic kidney disease – n (%) | 1 (3) |
| History of smoking – n (%) | 11 (37) |
| **Surgery type** |  |
| Coronary artery bypass grafting – n (%) | 17 (56) |
| Valve surgery – n (%) | 8 (27) |
| Combined procedures – n (%) | 5 (17) |
| **Extracorporeal Circulation** |  |
| Duration of extracorporeal circulation (min) | 81 [70-92] |
| Duration of aortic cross-clamp (min) | 60 [49-71] |
| **Blood gas analysis at admission to the ICU** |  |
| pH | 7.34 [7.32-7.36] |
| PaCO_2_ (kPa) | 5.6 [5.4-5.9] |
| PaO_2_ (kPa) | 14.5 [13.2-15.9] |
| PaO_2_/FiO_2_ | 266 [242-290] |
