# Supplementary material for: Six methods to determine expiratory time constants in mechanically ventilated patients: a prospective observational physiology study
Source: Intensive Care Med Exp. 2024 Mar 7;12:25. doi: 10.1186/s40635-024-00612-z (PMC10920606; doi:10.1186/s40635-024-00612-z)
Supplement: Supplementary file 4 — Additional file 4: Table S4. Measured first (τ1), second (τ2) and third (τ3) τ in PCV and VCV. [file 40635_2024_612_MOESM4_ESM.docx]

**Additional Table 4**: Measured first (***τ_1_***), second (***τ_2_***) and third (***τ_3_***) τ in PCV and VCV

| **PCV** | | | |  | **VCV** | | | |
| --- | --- | --- | --- | --- | --- | --- | --- | --- |
| *pt_id* | ***τ_1_*** *(s)* | ***τ_2_*** *(s)* | ***τ_3_*** *(s)* |  | *pt_id* | ***τ_1_*** *(s)* | ***τ_2_*** *(s)* | ***τ_3_*** *(s)* |
| 1 | 0.58 | 0.35 | 0.26 |  | 1 | 0.59 | 0.36 | 0.26 |
| 2 | 0.63 | 0.41 | 0.32 |  | 2 | 0.64 | 0.41 | 0.32 |
| 3 | 0.55 | 0.35 | 0.28 |  | 3 | 0.56 | 0.36 | 0.28 |
| 4 | 0.61 | 0.39 | 0.31 |  | 4 | 0.61 | 0.39 | 0.31 |
| 5 | 0.59 | 0.37 | 0.29 |  | 5 | 0.62 | 0.38 | 0.30 |
| 6 | 0.63 | 0.41 | 0.33 |  | 6 | 0.63 | 0.41 | 0.32 |
| 7 | 0.75 | 0.50 | 0.39 |  | 7 | 0.78 | 0.52 | 0.40 |
| 8 | 0.56 | 0.35 | 0.26 |  | 8 | 0.58 | 0.36 | 0.27 |
| 9 | 0.59 | 0.37 | 0.28 |  | 9 | 0.61 | 0.38 | 0.28 |
| 10 | 0.51 | 0.35 | 0.26 |  | 10 | 0.53 | 0.36 | 0.27 |
| 11 | 0.71 | 0.45 | 0.33 |  | 11 | 0.70 | 0.46 | 0.33 |
| 12 | 0.65 | 0.42 | 0.32 |  | 12 | 0.65 | 0.43 | 0.32 |
| 13 | 0.57 | 0.37 | 0.28 |  | 13 | 0.56 | 0.36 | 0.27 |
| 14 | 0.62 | 0.41 | 0.30 |  | 14 | 0.65 | 0.43 | 0.34 |
| 15 | 0.55 | 0.37 | 0.29 |  | 15 | 0.55 | 0.37 | 0.29 |
| 16 | 0.65 | 0.44 | 0.33 |  | 16 | 0.64 | 0.42 | 0.32 |
| 17 | 0.65 | 0.43 | 0.33 |  | 17 | 0.66 | 0.44 | 0.33 |
| 18 | 0.48 | 0.32 | 0.27 |  | 18 | 0.50 | 0.33 | 0.28 |
| 19 | 0.55 | 0.37 | 0.28 |  | 19 | 0.58 | 0.39 | 0.30 |
| 20 | 0.51 | 0.31 | 0.24 |  | 20 | 0.54 | 0.34 | 0.25 |
| 21 | 0.61 | 0.40 | 0.32 |  | 21 | 0.62 | 0.41 | 0.32 |
| 22 | 0.68 | 0.44 | 0.34 |  | 22 | 0.68 | 0.44 | 0.34 |
| 23 | 0.65 | 0.43 | 0.33 |  | 23 | 0.67 | 0.45 | 0.35 |
| 24 | 0.57 | 0.36 | 0.28 |  | 24 | 0.57 | 0.36 | 0.28 |
| 25 | 0.47 | 0.32 | 0.24 |  | 25 | 0.49 | 0.34 | 0.25 |
| 26 | 0.55 | 0.36 | 0.28 |  | 26 | 0.55 | 0.36 | 0.29 |
| 27 | 0.56 | 0.37 | 0.30 |  | 27 | 0.57 | 0.38 | 0.31 |
| 28 | 0.52 | 0.33 | 0.25 |  | 28 | 0.52 | 0.34 | 0.26 |
| 29 | 0.53 | 0.37 | 0.28 |  | 29 | 0.53 | 0.37 | 0.28 |
| 30 | 0.66 | 0.42 | 0.31 |  | 30 | 0.68 | 0.43 | 0.32 |
